# Balancing AI pedagogy and cross-disciplinary training for public health professionals

**DOI:** 10.1093/inthealth/ihag041

**Published:** 2026-05-12

**Authors:** Vikram Niranjan

**Affiliations:** School of Medicine, University of Limerick, Limerick V94 T9PX, Ireland; Health Research Institute, University of Limerick, Limerick V94 T9PX, Ireland

Dear Editor,

In our interconnected global village, diseases transcend borders with alarming speed—COVID-19’s rapid spread underscored this reality, infecting >700 million and claiming 7 million lives worldwide. As artificial intelligence (AI) emerges as a cornerstone of public health and global health practice, empowering healthcare professionals through balanced pedagogy and ethical training is imperative. This ensures they harness AI’s potential for surveillance, prediction and equity while mitigating risks like bias and data privacy breaches. Without such preparation, we risk exacerbating inequities in low-resource settings.^1^

Public health professionals must master AI not as technologists, but as informed integrators who prioritize human-centred applications. Traditional training often silos AI within computer science, neglecting public health curricula. A balanced pedagogy fuses epidemiology, ethics and data science: core modules on machine learning for outbreak forecasting (e.g. random forest plots analysing syndromic data), alongside ethical frameworks like the World Health Organization’s AI principles emphasizing transparency and accountability. Cross-disciplinary exposure—pairing clinicians with data scientists, ethicists and policymakers—fosters innovative problem-solving. For instance, hybrid courses could simulate real-time antimicrobial resistance (AMR) surveillance in sub-Saharan Africa (SSA), drawing from genomic datasets and local contexts, as highlighted in recent reviews.^2^

Why empower professionals now? Global disease dynamics demand it. Pathogens like Mpox or novel influenza variants spread via air travel and trade, necessitating AI-driven tools for real-time genomic sequencing and contact tracing. Yet untrained users may amplify biases; an AI model trained on Global North data fails in diverse SSA populations, widening health disparities.^3^ Ethical AI use—via explainable models and federated learning—ensures fairness, protecting vulnerable groups in our global village. Professionals trained thus become stewards, auditing algorithms for equity and integrating One Health perspectives linking human, animal and environmental data.

This training directly combats global health inequity by equipping professionals in low- and middle-income countries to adapt AI tools locally, countering the ‘digital divide’ where 90% of health AI research originates from high-income nations. Federated learning enables secure data pooling without centralization, allowing SSA clinicians to refine models with indigenous datasets for precise AMR predictions, averting 1.27 million annual deaths. Cross-disciplinary pedagogy fosters South–South collaborations, scaling AI for maternal health in rural India or vaccine equity in Ethiopia. Ethical oversight prevents discriminatory algorithms, ensuring AI amplifies marginalized voices—reducing neonatal mortality gaps by 20% through predictive analytics. Ultimately, empowered professionals democratize AI, helping to narrow the growing economic burden of health inequities projected to reach trillions by 2040 and promoting universal access in our borderless world.

Innovative training pathways amplify this. Micro-credentials via third-level universities or online platforms specialize skills without full degrees. Immersive simulations using agentic AI mimic pandemics, teaching decision-making under uncertainty. Cross-disciplinary ‘AI health labs’ in universities, akin to Ireland’s Health Innovation Hub,^4^ rotate professionals through bioinformatics, policy and community engagement. Global consortia standardize curricula, ensuring scalability from one continent to another.^5–7^

Empowering professionals safeguards against AI pitfalls: overreliance erodes clinical judgment, while unregulated tools invite surveillance overreach. Rationales rooted in globalization—trade, migration, climate-driven outbreaks—compel ethical mastery. Trained experts can deploy AI chatbots for multilingual health messaging or predictive analytics for vaccine allocation, averting crises proactively.

This agenda aligns with United Nations Sustainable Development Goals (SDGs): SDG 3 (Good Health and Well-being) via AI-enhanced disease prevention, SDG 4 (Quality Education) through inclusive pedagogy, SDG 10 (Reduced Inequalities) by narrowing digital health gaps and SDG 17 (Partnerships for the Goals), fostering cross-disciplinary collaborations.

International Health could lead by advocating AI-integrated public health courses, urging global aid funders to prioritize pedagogy grants. As we navigate the AI surge, balanced training equips public health professionals to thrive ethically, fortifying our global defences.

## Data Availability

No new data were generated or analysed in support of this research.
